# From gut microbiota to brain: implications on binge eating disorders

**DOI:** 10.1080/19490976.2024.2357177

**Published:** 2024-05-23

**Authors:** Weiwei Guo, Wei Xiong

**Affiliations:** a Department of Neurology, The First Affiliated Hospital of USTC, Division of Life Sciences and Medicine, Hefei National Research Center for Physical Sciences at the Microscale, University of Science and Technology of China, Hefei, China; b Anhui Province Key Laboratory of Biomedical Imaging and Intelligent Processing, Institute of Artificial Intelligence, Hefei Comprehensive National Science Center, Hefei, China; c CAS Key Laboratory of Brain Function and Disease, Hefei, China

**Keywords:** Gut-brain-axis, eating disorders, binge eating

## Abstract

The prevalence of eating disorders has been increasing over the last 50 years. Binge eating disorder (BED) and bulimia nervosa (BN) are two typical disabling, costly and life-threatening eating disorders that substantially compromise the physical well-being of individuals while undermining their psychological functioning. The distressing and recurrent episodes of binge eating are commonly observed in both BED and BN; however, they diverge as BN often involves the adoption of inappropriate compensatory behaviors aimed at averting weight gain. Normal eating behavior is coordinated by a well-regulated trade-off between intestinal and central ingestive mechanism. Conversely, despite the fact that the etiology of BED and BN remains incompletely resolved, emerging evidence corroborates the notion that dysbiosis of gastrointestinal microbiome and its metabolites, alteration of gut-brain axis, as well as malfunctioning central circuitry regulating motivation, execution and reward all contribute to the pathology of binge eating. In this review, we aim to outline the current state of knowledge pertaining to the potential mechanisms through which each component of the gut-brain axis participates in binge eating behaviors, and provide insight for the development of microbiome-based therapeutic interventions that hold promise in ameliorating patients afflicted with binge eating disorders.

## 1. Introduction

Eating disorders (EDs) are a group of distinctive mental illnesses characterize by appetite and weight disturbance accompanied with substantial behavior and psychological impairments. The prevalence of EDs in Western settings for the years 2013–2022 is 5.5–17.9% in young women and 0.6–2.4% in young men,^1^ with each year an estimated 3.3 million healthy person-years were lost worldwide.^2^ Binge eating disorder (BED) and bulimia nervosa (BN) are two classical forms of EDs that were included into the Diagnostic and Statistical Manual of Mental Disorders (DSM) and the International Classification of Diseases (ICD) in 2013 and 2019, respectively.^3,4^ The 2022 epidemiological statistics revealed that the prevalence of lifetime DSM-5 BED ranged from 0.6% to 6.1% among women and from 0.3% to 0.7% among men in Western countries, while BN was reported by 0.8% to 2.6% of women and by 0.1% to 0.2% of men.^1^ Emerging researches from Eastern Europe, Asia and Latin America have also indicated similar high prevalence. Even during the COVID-19 pandemic, the incidence of BN and BED has continued to escalate. The core psychopathological characterization of both BED and BN lies in the recurrent indulgence in binge eating episodes, wherein individuals consume significantly larger quantities of food than usual within a discrete timeframe, accompanied by an overwhelming sense of losing control over their eating behavior. The regular utilization of inappropriate compensatory purging behaviors, such as excessive physical exertion, self-induced emesis, or laxative misuse, in order to maintain body weight, however, constitutes a diagnostic symptom for BN.^5^ In contrast, individuals with BED do not have regular compensatory behaviors after recurrent binge eating. Studies have demonstrated that both BED and BN can give rise to grave medical complications. For instance, both BED and BN are often associated with or lead to obesity (30–45%),^6,7^ diabetes^8^ and other related metabolic syndromes. Around 50% of patients with BED or BN co-occurred with attention deficit hyperactivity disorder, and about 15% of patients exhibited multiple comorbid impulsive behaviors including alcohol and substance abuse, compulsive buying and multiple sexual relationships.^9^ Moreover, BED and BN also frequently co-occur with at least one additional mental health condition, encompassing mood disturbance, post-traumatic stress disorder and borderline personality disorder.^9^ Considering the global prevalence and profound health ramifications of BED and BN, researchers have dedicated themselves to unraveling the etiology and pathogenesis of binge eating over the past few decades.

Trillions of microorganisms including bacteria, archaea, fungi and virus reside in the gastrointestinal tract to form an ecological community called the gut microbiome. The field of microbiome research has witnessed an extraordinary surge in the past few decades, with a growing consensus that the gut microbiome plays a profound role in maintaining both physical well-being and mental health.^10,11^ As mental disorders, BED and BN involve compromised brain regions and neurocircuitry encoding appetite control, food craving, rewarding processing and impulsivity.^8^ This concept prompts a flood of contemporary research that seeks to explore how gut microbiome acts in conjunction with the brain to ultimately influence various aspects of binge eating-related behaviors. The microbiota communicate with the brain via both “direct” and “indirect” pathways,^11^ including hundreds of metabolites, neuronal connections, chemical transmitters and the immune system. Conversely, signaling from the brain through the hypothalamic-pituitary-adrenal (HPA) axis and the autonomic nervous system exerts influence over gut microbiome composition as well as gastrointestinal function.^11^ In fact, emerging evidence suggests the bidirectional communication within the microbiota-gut-brain axis plays a pivotal role in the pathophysiology of binge eating disorders, with both gut to brain and brain to gut signaling mechanism implicated.^12–14^ In this review, we endeavor to present a comprehensive overview of recent advancements concerning the involvement of the gut microbiota and the brain, as well as their intricate connections in binge eating. Furthermore, we aim to provide valuable insights into potential therapeutic and diagnostic options that target each level of the microbiota-brain axis, thereby augmenting existing interventions.

## 2. Diagnostic criteria and clinical manifestations of BN and BED

According to DSM-5 and the ICD-11, BN is characterized by recurrent episodes of indulging in excessive amounts of food.^3,4^ In DSM-5, a binge eating episode is defined as consuming objectively larger quantities of food than within a relatively short timeframe than what would typically be consumed by most individuals.^3^ In addition, DSM-5 specifies the presence of an overwhelming sense of “lack of control” over one’s choice and quantity of food during the esipode.^3^ ICD-11 defines the binge eating episode as a distinct period of time when an individual experiences a subjective loss of control over their consumption, indulging significantly more or differently than usual.^4^ Both criteria involve the presence of inappropriate compensatory behaviors (e.g., self-induced vomiting; fasting; excessive exercise; misuse of diuretics, laxatives or other medications) in order to thwart any potential weight gain. Meanwhile, individuals with BN are preoccupied with weight and body shape, which unduly exert influence on their self-evaluation.

Resembling BN, both DMS-5 and ICD-11 delineate BED as distressing, recurrent episodes of indulging in copious amounts of food when not experiencing physical hunger, consuming at an accelerated pace, continuing until uncomfortably satiated, and subsequently experiencing subjective negative emotions such as repulsion or remorse. It is worth noting that unlike in BN, the binge eating episodes associated with BED are not regularly succeeded by inappropriate compensatory behaviors.

## 3. Gut microbiome dysbiosis in BED and BN

The composition of gut microbiome is notably influenced by dietary changes^14,15^ and stress,^16^ which are the two primary contributors that trigger and prolong binge-eating behaviors.^17^ Most animal models of binge eating also incorporate elements associated with the human condition, such as additional environmental stressors and dietary modifications (e.g., restriction/refeeding paradigms, compensatory access to highly palatable food and beverages). The heightened utilization of antibiotics in patients with BED and BN prior to the emergence of binge eating symptoms signifies preexisting dysbiosis in their gut microbiome.^18^ Despite the relative scarcity of studies on gut microbial diversity and taxonomic disparities among individuals exhibiting binge eating behaviors, available evidence suggests a tendency toward increased Firmicutes and Enterobacteriaceae, as well as decreased α diversity in BED.^19^ A small-scale empirical investigation was conducted, utilizing 16S rDNA sequencing to compare the gut microbiome composition in fecal samples obtained from 42 patients with both obesity and BED, with samples collected from 59 patients who were obese but did not have BED.^20^ In this study, individuals with BED displayed up-regulated levels of *Anaerostipes*, *Roseburia* and *Bifidobacterium* as well as down-regulated levels of *Akkermansia* and *Intestinimonas* in comparison to those without BED. Another study delved into the disparities in gut microbiome profiles between individuals with restricting anorexia nervosa (ANR) and those with binge-purging AN (ANBP), revealing significantly elevated relative abundances of *Eubacteriacae* and *Bifidobacteriaceae*, alongside a diminished presence of *Pasteurellaceae* in ANBP subjects compared to their ANR counterparts.^21^ Additionally, a recent study explored the gut microbiome profiles of 21 anorexia nervosa subjects, 9 BED subjects, 17 BN subjects and 28 healthy controls. The findings revealed that patients who restrict their food intake exhibited a relatively high abundance of *Bacteroides*, while those who engage in binge-purging behaviors were characterized by *Prevotella*. In contrast, the healthy controls showed higher abundance of *Bifidobacterium* and *Collinsella*.^22^

Our latest investigation into the alterations of gut microbial communities in mice exhibiting binge eating-like behaviors, utilizing 16S rDNA sequencing, revealed an augmented relative abundance of *Bacteroidaceae* and *Lachnospiraceae*, alongside a diminished presence of *Lactobacillaceae* and *Ruminococcaceae* when compared to their control littermates. At the genus level, we observed an augment of *Bacteroides*, *Roseburia* and *Alistipes*, as well as a loss of *Lactobacillus* and *Ruminococcaceae-UCG-014* in binge mice.^12^ Meanwhile, an analysis of gut microbiota composition of 11 female patients diagnosed with BN revealed a significant reduction in the abundance of *Faecalibacterium*. Variable importance in projection score plot further indicated that *Faecalibacterium prausnitzii*, belonging to the genus *Faecalibacterium*, made the most significant contribution to the discrepancy between BN subjects and healthy controls.^12^ Considering the heterogeneity of findings across various investigations on dysbiosis in binge eating, potential factors contributing to this variability may encompass disparities in study design, methodology, or individual variations among patients with binge eating disorders. Although the significance of these gut microbiome changes can be speculated, replicated studies in larger well-characterized samples are necessary before definitive conclusions can be drawn. A study called “The Binge Eating Genetics Initiative (BEGIN)”, released in 2020, endeavors to further augment the comprehension surrounding the pathogenesis of BED and BN. The researchers plan to recruit 1000 subjects diagnosed with BED and BN based on DSM-5 criteria, in order to examine the interplay between gut microbiota, genomic factors, and behavioral patterns in these disorders.^23^ Still, to date the research of the gut microbiome in binge eating-related disorders is in its infancy and calls for further in-depth investigation.

## 4. The microbiota-gut-brain axis in binge eating: state of the art

The mounting evidence has revealed a mutually influential connection between alterations in the intestinal microbiome and the manifestation of binge eating behaviors. First, shifted dietary patterns of individuals with BED and BN can lead to disruption in the composition of gut microbial communities. Second, dysbiosis of the gut microbiota may further contribute to enduring symptoms of binge eating and other associated comorbidities. Whether as a causative factor or an adaptive mechanism, currently, several hypothesizes have been proposed regarding the potential role of the gut microbiome in binge eating by influencing host appetite, food choice and mood regulation through the microbiota-gut-brain axis encompassing metabolic, humoral, immune and neural pathways.^8,24^

### 4.1. Metabolic/humoral pathway

#### 4.1.1. Short-chain fatty acids

Short-chain fatty acids (SCFAs), such as acetate, propionate and butyrate, are important bacterial metabolites produced by the gut microbiome via fermentation of indigestible carbohydrates.^25^ The levels of SCFAs were found to be significantly elevated in individuals who are obese or overweight.^26^ The appetite-modulating functions of SCFAs are exerted by their binding to G-protein-coupled receptors, including free fatty acid receptor 2 (FFAR2, GRP43) and free fatty acid receptor 3 (FFAR3, GRP41), which are expressed in various tissues and organs.^27^ Interestingly, the activation of various FFARs elicits disparate effects on the host’s appetite. For instance, SCFAs activate ghrelin-related signaling by acting on FFAR3 in islets, inhibiting insulin secretion and subsequently promoting appetite.^28,29^ Conversely, SCFAs can also suppress appetite through their binding to FFAR2 and subsequently inducing the release of peptide tyrosine-tyrosine (PYY), glucagon-like peptide-1 (GLP-1), insulin and leptin.^30–32^ The gut microbiota-derived SCFAs, when released into the bloodstream, have the remarkable ability to traverse the blood–brain barrier (BBB) and directly exert their effects on appetite-related neurons within the central nervous system.^33^ Frost et al. found that^13^C acetate produced by fermentation of^13^C-labeled carbohydrate in the colon elevates hypothalamic^13^C acetate higher than baseline level. In addition, intraperitoneal injection of the SCFA acetate reduced acute food intake by inducing an anorectic neuropeptide expression profile in the hypothalamus, including up-regulation of pro-opiomelanocortin (POMC) and down-regulation of agouti-related peptide (AgRP).^33^ Moreover, SCFAs have been reported to attenuate ghrelin-mediated signaling via binding to growth hormone secretagogue receptor (GHSR)-1a, thereby indirectly altering motivation and reward.^34^ Collaboratively, the metabolites of microbiota known as SCFAs were implicated in regulating appetite through the gut-brain axis.

#### 4.1.2. Appetite-related peptides

Various appetite-regulating neuropeptides are synthesized in the gastrointestinal tract and play indispensable roles in the pathology of binge eating disorders. Appetite-suppressing peptides include PYY, GLP-1, α-melanocyte-stimulating hormone (α-MSH), cholecystokinin (CCK), and appetite-stimulating hormones such as neuropeptide Y (NPY) and ghrelin. In individuals of optimal health, the plasma concentration of ghrelin exhibited an inverse correlation with body mass index (BMI),^35^ while PYY also demonstrated a negative association with body weight.^36^ However, two independent research groups reported that patients with BN, despite having a higher BMI, exhibited elevated plasma ghrelin concentrations before food intake, and a diminished response of ghrelin following food ingestion.^37,38^ The rise in plasma PYY following food intake was also blunted in those BN patients. The altered response of ghrelin and PYY to food ingestion may contribute to the perpetuation of binge eating behaviors in BN patients. Ghrelin exerts its regulatory control over appetite and energy homeostasis through GHSR-1a or vagal afferents. Meanwhile, ghrelin suppresses the activity of α-MSH-secreting neurons, thereby enhancing inhibitory GABAergic projections from NPY/agonist-related peptide (AgRP) neurons to hypothalamic arcuate glutamatergic neurons and ultimately resulting in an elevation in energy intake.^39^ Notably, the gut microbiome is also capable of synthesizing molecules that exhibit analogous sequence and conformational homologies to specific neuropeptides.^40^ For instance, caseinolytic protease B homologue protein (ClpB) produced by *Escherichia coli* (*E. coli*) is a well-known mimetic of α-MSH and is capable of exerting α-MSH-like functions, such as promoting secretion of PYY and GLP-1, and directly activating anorexigenic neurons to induce satiety.^41,42^ Elevated plasma levels of bacterial ClpB protein have been detected in patients with EDs including anorexia nervosa, BN and BED.^43^ Besides “mimicking” α-MSH, ClpB can provoke the synthesis of α-MSH autoAbs.^44^ For example, a study revealed elevated levels of IgG autoAbs against α-MSH in bulimic patients.^45^ In this study, the authors revealed a novel functional role of α-MSH autoAbs, demonstrating that instead of neutralizing their target as traditionally believed, these natural autoantibodies enhance α-MSH signaling through the MC type 4 receptor.^45^ The significant correlation observed between plasma levels of α-MSH reactive autoAbs and the severity of psychopathologic features, as measured by the EDI-2 scale, underscores their clinical relevance to eating disorders.^46^ In fact, IgG and IgA autoantibodies against various appetite-regulating peptides were detected in plasma from both healthy individuals and patients with BN.^44,46^ Fetissov et al. showed a close correlation between the levels and affinities of these autoantibodies and psychological traits in patients with eating disorders, suggesting their potential as significant contributors to the mechanisms governing motivation for binge eating.^46^

#### 4.1.3. Neurotransmitters

As a category of intestinal microbial metabolites, neurotransmitters may also play a role in the mechanisms underlying binge-eating disorders. GABA produced by intestinal microbiota can act as neurotransmitter to engage in communication between gut and brain. Mechanistically, GABA is a widely recognized molecular signal in regulating gastrointestinal motility and secretion of various appetite-controlling neuropeptides.^47^ Disruption of GABAergic signaling attenuates NPY-induced hyperphagia and hunger-driven feeding.^48^ In patients with anorexia nervosa, Prochazkova et al. discovered a reduction in GABA concentration within their fecal matter;^49^ however, current research on the direct correlation between the gut microbiota-derived GABA and binge eating remains limited. GABA functions as an inhibitory neurotransmitter in the central nervous system (CNS). Zhang et al. reported that acute activation of GABAergic neurons in zona incerta (ZI) or their projection terminals to paraventricular thalamus (PVT) induced rapid binge-like eating behaviors in mice, whereas chronic activation of inhibitory ZI-PVT circuit leaded to persistent overeating and weight gain.^50^ Still, whether gut-derived GABA can cross the BBB to specific brain regions and act on targeted neurons to regulate feeding behaviors requires further investigation.

5-HT is another neurotransmitter produced by host intestinal enterochromaffin cells (ECs) and the brain, with ECs accounting for 95% of body’s production of 5-HT.^51^ Increased peripheral 5-HT level was associated with obesity.^52^ Furthermore, 5-HT has been implicated in suppressing appetite and satiety control by exerting its influence on various aspects of intestinal function, including sensory perception, nutrient absorption, secretion dynamics, and gastrointestinal motility.^53–55^ The direct passage of 5-HT across the BBB may be limited, but the transportation of 5-HT by circulating platelets to the brain and subsequent elevation of CNS 5-HT levels establish a crucial connection between intestinal 5-HT and cerebral functions.^56^ Selective activation of 5-HT_2c_R-positive neurons in the nucleus of the solitary tract (NTS) within the brainstem resulted in a significant reduction in food intake.^57^ The 5-HTergic neurons located in the dorsal raphe nucleus (DRN) were also reported to regulate distinct types of feeding behavior through parallel downstream projection circuits. Activation of 5-HTergic projections from DRN to hypothalamic arcuate nucleus (ARH) exerts inhibitory control over food intake driven by hunger, mediated through 5-HT_2c_R and 5-HT_1B_R within the ARH. Conversely, activation of DRN 5-HTergic projections to the ventral tegmental area (VTA) dampens hedonic feeding via 5-HT_2c_R.^58^ Animal study conducted by Price et al. showed that activation of 5-HT_2c_R relieved the binge intake of high-fat food.^59^ Another research group has also showcased the role of 5-HT_2c_R in mice’s binge eating behavior.^60^ Activation of 5-HT_2c_R populations in dopamine neurons effectively inhibits binge eating-like behaviors in intermittent high-fat diet mice. Furthermore, numerous studies have elucidated the aberrations within the 5-HTergic system in patients exhibiting binge eating behaviors. For instance, the role of L-tryptophan, a 5-HT precursor, was significantly diminished possibly due to dysfunction of tryptophan hydroxylase-2, the enzyme responsible for synthesizing 5-HT in the brain.^61^ During fasting periods, 5-HT levels in patients exhibited a more pronounced decrease compared to the control group, consequently giving rise to episodes of irritability and indulgence in binge eating.^62^ In addition to regulating food intake, 5-HT also has mood-modulating and anxiety-relieving effect. As aforementioned, individuals with BED or BN exhibited higher prevalence of neuropsychiatric comorbidities including anxiety and depression. The persistent of impaired 5-HTergic signaling during remission of BN syndrome raises the possibility that it may have been one of the contributing factors preceding the onset of binge eating.^63^ In fact, BED patients were reported to exhibit a more impoverished mood state prior to binge eating and tend to experience a greater burden of negative emotions compared to those without BED.^64^ In BN women, acute tryptophan depletion leads to a more pronounced elevation in peak depression, heightened susceptibility to mood fluctuations, and an intensified craving for binge eating compared to healthy controls.^65^ Notably, in accordance with international guidelines, second-generation antidepressants known as selective serotonin reuptake inhibitors (SSRIs) have been recommended as a pharmacotherapy option for addressing binge eating disorders^8^; although available clinical data suggests that the amelioration of binge symptoms diminishes after 3–6 months.^66^ Together, these findings indicate the potential involvement of gut microbiota and brain-derived 5-HT in regulating appetite and energy intake, thereby prompting further exploration into the intricate mechanisms by which the 5-HTergic system contributes to binge eating development.

### 4.2. Immune pathway

Aside from metabolites, lipopolysaccharides (LPS), the gut bacterial product, presents itself as an alternative mediator for gut microbial regulation of host appetite and cognitive function via the gut-brain axis. When binding with Toll-like receptors (TLR) expressed in enteroendocrine cells, LPS affects the release of neuropeptides that govern appetite and satisfaction.^67^ The stimulation of LPS prompts immune cells to secrete a cascade of cytokines (predominantly IL-1, IL-6 and TNF-α) and trigger a series of alteration in the immune-endocrine-nervous system, which in turn activate the HPA axis that regulate host response to both physical and psychological stressors and is fundamental to development and progression of eating disorders.^68^ Animal studies demonstrated that LPS initiates an anorexic response by directly stimulating TLR-4/MyD88 signaling pathway in the CNS.^69,70^ In addition, LPS increases the BBB permeability by exerting its influence on tight junction proteins and interacting with brain endothelial cells, thereby affecting circulating cytokines transport and immune cell migration. This may serve as an indirect mechanism through which gut microbiota modulates central appetite.^71^

### 4.3. Vagal pathway

It is widely acknowledged that the gut and the CNS can communicate bidirectionally through autonomic nervous system. A subset of gut enteroendocrine cells sense nutrients and other chemical stimuli directly, transmitting these intestinal signals to vagal neurons through serotonergic or glutamatergic synapses.^72,73^ GPR65-expressing vagal afferent neurons extend terminals into intestinal villi of mice and respond to enteric nutrients,^74^ whereas GLP-1 receptor-expressing mechanosensing vagal afferents reach out toward muscle layers in order to detect gastrointestinal stretch.^74,75^ Recent studies have demonstrated novel functions of the vagus nerves in regulating feeding control and determining nutrient preferences through central neural circuits involving the nucleus of the solitary tract (NTS) and downstream projection regions. The close proximity of vagal afferent neurons’ nerve endings to the gut microbiota in the gastrointestinal lumen at one end, coupled with their connection to the central nervous system at the other, renders the vagal pathway a pivotal constituent of the gut-brain axis for regulating host ingestive behavior.

Vagal neurons express a diverse array of G protein-coupled receptors (GPCRs) and can be categorized based on the expression of genes encoding these receptors, such as Gpr65, Gpr174, Htr3a/b, Piezo1, Cysltr2, Ntsr1, and S1pr3.^76^ High-throughput screening of microbial ligand-receptor bindings has revealed evidence of interactions between gut microbiome metabolites and some of these GPCRs, such as GPR35, Gpr149, Gpr174, Ntsr1, and S1pr3.^77,78^ Johnson et al. reported that sphingolipids produced by gut microbiota can bind to S1pr3.^79^ In addition, it has been reported that aromatic acidic metabolites, such as the tryptophan derivative kynurenic acid (KYNA), possess the ability to bind with GPR35.^72^

A recent study demonstrated a decline in luminal KYNA levels in mice exhibiting binge-eating like behaviors, alongside diminished gut KYNA levels observed in BN patients. The chronic oral administration of KYNA significantly relieved the excessive preference for palatable food and reduced the total calorie intake of binge mice.^12^ The vagus nerve endings in the ileum express dense N-methyl-D-aspartate receptors (NMDARs). Optimal concentrations of KYNA within the gastrointestinal tract bind to these NMDARs located at the vagus nerve terminals, thereby modulating food intake and maintaining energy equilibrium. In contrast, the absence of gut probiotics and fluctuation in luminal KYNA disinhibit the vagus nerve within the gastrointestinal tract, leading to subsequent hyperactivation of the gut-brain axis and manifestation of binge eating syndrome in both individuals with BN and mice exhibiting binge-modeling behavior.^12^

Besides KYNA, various other microbiota metabolites such as bile acids, indoles and SCFAs have also been documented for their ability to interact with vagal neurons or intestinal enteroendocrine cells to regulate feeding behaviors. The bile acids synergized with CCK to enhance satiety via activation of vagal afferent pathways,^80,81^ while indoles induce GLP-1 secretion by enteroendocrine L-cells, further stimulating colonic vagal afferent activities.^82^ Similarly, SCFA propionate can also elicit GLP-1 and PYY release from enteroendocrine cells and colonic crypts via FFAR2 or GPR43.^83,84^ Treatment of enteroendocrine cells line with propionate or butyrate leads to elevated mRNA levels of umami taste receptors, this effect was reversed upon inhibition of G αi/o signaling, suggesting that SCFAs shape enteroendocrine sensitivity to bioactive nutrients via FFAR2/3.^85^ Additionally, SCFAs have been reported to directly act on vagal neurons expressing SCFA receptor FFAR3 or GPR41, thereby regulating the process of food ingestion.^77^ Intraperitoneal administration of SCFAs, especially butyrate, exerts a significant suppressive effects on food intake in fasted mice. This effect can be mitigated by the denervation of capsaicin-induced sensory nerves and the hepatic vagotomy.^86^ The impact of butyrate on the gut-brain axis includes the activation of intracellular Ca^2+^ signaling in isolated vagal neurons, as well as the phosphorylation of cellular activation markers ERK1/2 in nodose ganglion and medial region of NTS.^86^

**Figure 1.**
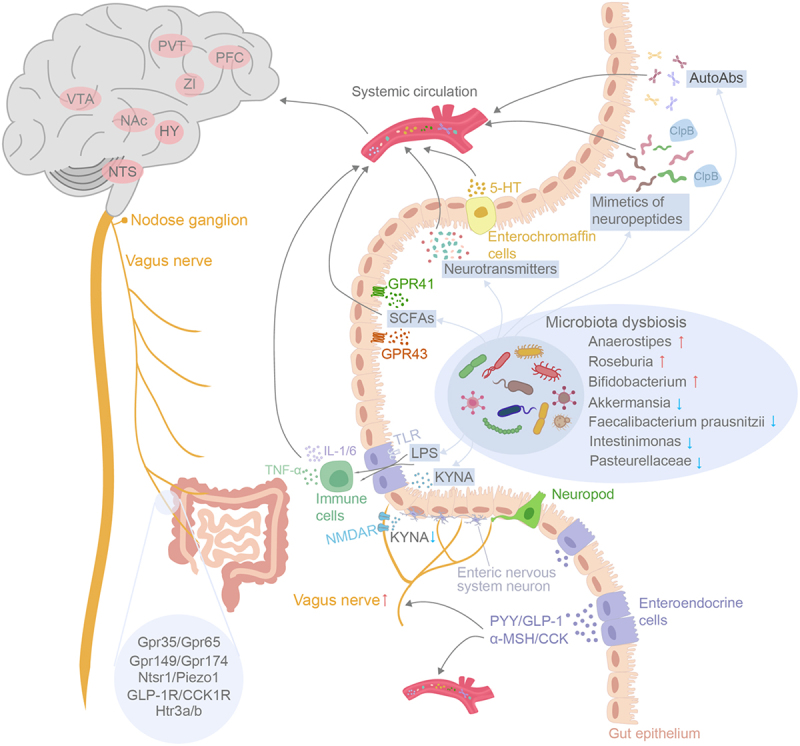
Schematic outline depicting the intricate pathways of gut-to-brain communication in binge eating disorders. The routes involve metabolic, humoral, endocrine, immune and neuronal pathways. BED and BN were associated with microbiota dysbiosis within the gut. The diverse array metabolites produced by the gut microbiome such as SCFAs, neurotransmitters, neuroactive peptides, can travel through systemic circulation to the brain to modulate host appetite indirectly. SCFAs also possess the capability to stimulate enteroendocrine cells into releasing gut hormones such as PYY, GLP-1 and CCK, which effectively regulate appetite and food intake either through systemic circulation or by acting upon afferent pathway of the vagus nerves. The gut microbiota-derived LPS influences BBB permeability by activating the immune response, ultimately leading to disruption of the host’s energy homeostasis. Finally, the diminished KYNA release from gut microbiota binds to NMDARs and triggers the onset of binge eating syndrome by directly activating the vagus-NTS-PVT neuronal pathway. In CNS, the alteration of various brain nuclei functions has been associated with binge eating behavior, including NTS, hypothalamus (HY), nucleus accumbens (NAc), VTA, Zona incerta (ZI), prefrontal cortex (PFC) and PVT.

In conclusion, maintaining a harmonious gut microbiome is crucial for the host to uphold a healthy appetite and eating behavior. The disruption of intestinal microbiota balance contributes to the development of binge eating disorders by perturbing various metabolic, humoral, endocrine, immune, and neuronal pathways within the gut-brain axis (Figure 1). Consequently, restoring microbial equilibrium and optimizing gut-brain axis functionality could potentially serve as viable therapeutic targets for BED and BN.

## 5. Brain regions and circuits involved in the pathopsychology of binge eating

### 5.1. Feeding control

Once entering the CNS, the vagal gastrointestinal afferent neuron endings initially terminate within the brainstem’s nucleus of NTS,^87^ and form multisynaptic circuits via the NTS to high-order brain regions related to feeding control and reward, such as PVT,^12^ arcuate nucleus (ARC)^88^ and striatum.^89^ In a model of mice experiencing an excessive bout of overeating, the c-fos expression in the NTS, PVT, ARC, the dorsal medial hypothalamus and other nuclei associated with feeding or stress exhibited a significant elevation.^12^ Upon optogenetic activation of the PVT glutamatergic neurons, mice exhibited robust food-seeking behavior and an increased preference for palatable food and calorie intake, whereas functional inhibition of these neurons considerably reversed the excessive inclination toward palatable food consumption.^12^ Brain circuits tracing utilizing retrograde pseudorabies virus PRV-EGFP has revealed a vagus nerve-NTS-PVT pathway. Chemogenetic inhibition of this pathway or subdiaphragmatic vagotomy can attenuate binge eating-like behaviors in mice, including a diminished preference for delectable food and reduced calorie intake. These findings offer compelling evidence that dysfunction of the intestinal vagus nerve-NTS-PVT neural pathway constitutes, at least in part, a potential mechanism underlying the manifestation of binge eating behavior in mice.

### 5.2. Motivation

Motivation for food seeking is an intense longing to partake in the consumption of nourishment, particularly delectable fare. The corticostriatal circuitry orchestrates motivated behaviors in response to rewarding stimuli, encompassing both delectable food and valuable currency. Specifically, circuit networks comprising NAc, VTA, lateral hypothalamus, amygdala (Amy) and orbitofrontal cortex (OFC) are involved in the intricate dance of motivation underlying food craving. Evidence from multiple neuroimaging studies on the neural activities in central brain regions of patients with BED consistently indicate the neuroanatomical and functional maladaption of corticostriatal circuitry.^90,91^ The hyperactivities in striatal regions of BED patients are associated with dopaminergic signaling, which triggers an insatiable desire for food akin to that observed in individuals grappling with substance abuse. In both cross-sectional and prospective studies, there has been a strong correlation observed between food craving and the frequency as well as severity of binge eating episodes.^92,93^

### 5.3. Impulsivity and decision-making

The trait of impulsivity manifests as an inclination toward heightened drives for rewards, prompting impulsive actions devoid of adequate contemplation of potential drawbacks. Individuals with BED were found to exhibit elevated impulsivity scores on the UPPS and Barratt (BIS-11) impulsiveness scale, diminished self-control, as well as impaired set-shifting indicative of perseverance/compulsive behaviors when compared to non-BED obese and normal weight subjects.^94–97^ Several brain structures, including the striatum, hippocampus (Hipp), PFC, and anterior cingulate cortex (ACC) were involved in impulsivity control.^94^

Decision-making is a complicated cognitive process that generally involves goal-directed and habitual components, which ultimately culminating in the choice of one outcome from among various options. Both the dorsomedial and dorsolateral part of striatum were critical for the conscious and habitual decision-making.^98^ In tasks involving the selection between risky or certain monetary options, obese individuals with BED exhibited compromised decision-making, as evidenced by a heightened propensity to opt for risky choice characterized by moderate reward probabilities and high loss probabilities compared to non-BED obese subjects and healthy volunteers.^99,100^ The ability to resist the allure of immediate but smaller rewards in favor of obtaining larger incentives at a later time, known as delay discounting, represents yet another dimension of decision-making. In individuals with BED who are obese, there is a notable decrease in their inclination toward delayed and probabilistic rewards.^101^ Additional studies also discovered a connection between high delay discounting rates and overeating disorders,^102,103^ as well as particular personality traits such as impulsivity^103,104^ and other neuropsychiatric conditions.^105^

### 5.4. Execution

**Figure 2.**
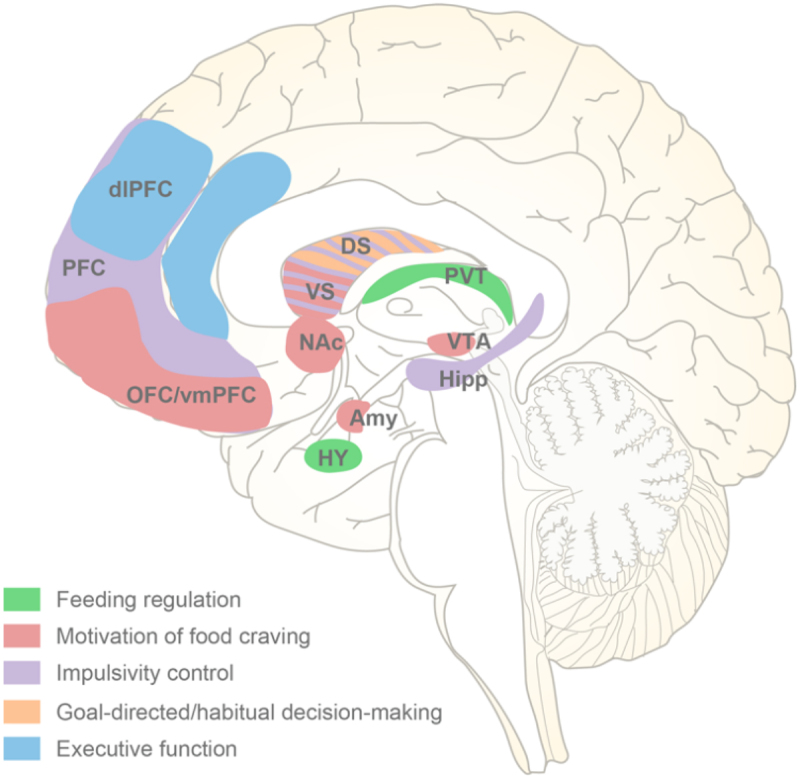
Brain regions in the pathopsychology of binge eating. Based on multiple neuroimaging studies, the central neurological risk factors implicated in binge eating episodes include the dysfunction of five network systems that regulate feeding, motivation, impulsivity control, decision-making and execution. DS, dorsal striatum; VS, ventral striatum.

Execution refers to a group of sophisticated cognitive faculties that enable a person to empower individuals to proficiently execute intricate daily tasks. The prefrontal cortex, especially its dorsolateral subregion (dlPFC), is recognized as the major neural substrate for execution.^106^ The presence of deficits in working memory, cognitive flexibility, and inhibitory control, which are the three fundamental components of executive function, has been found to contribute to symptoms associated with binge eating.^107,108^ Decreased inhibitory control is related to hypoactivities of the insula, the ventromedial prefrontal cortex (vmPFC), and inferior frontal gyrus in BED subjects.^109^ Additionally, the alterations of reward processing may also play a role in the initiation and maintenance of binge eating.^110^ Leenaerts et al. systematically reviewed the resting structural and functional changes of brain rewarding system under binge eating conditions, and found that individuals with binge eating syndrome had higher volume of the cortical areas including the OFC, insula and ACC, as well as lower striatal dopamine transmission and functional connectivity between striatum and the frontal cortex^111^(Figure 2).

## 6. Gender difference in the prevalence of binge eating disorders

Due to social, cultural, and other challenges, men with EDs generally face a perceived social stigma. In studies involving face-to-face interviews on eating disorders, it was observed that men tended to exhibit heightened levels of embarrassment or hesitation when acknowledging their struggles compared to women.^112^ Additionally, they often held a less sympathetic perspective toward individuals with eating disorders, perceiving them as not posing a significant health concern. Epidemiological evidence also suggests that the prevalence of binge eating disorders is lower among boys and men in comparison to girls and women.^1^ Indeed, the female-to-male ratios for BED and BN range from 2:1 to 10:1.^3^ Although psychosocial factors, such as societal pressure on women’s appearance and weight, contribute to gender disparities in binge eating disorders; nevertheless, research suggests that biological factors also play a pivotal role. Culbert summarized compelling evidence supporting the intricate biological mechanisms underlying the sex-specific prevalence of eating pathology.^113^ The primary contributors that influence the differential risk for eating pathology across genders throughout the lifespan are sex steroid hormones, including testosterone, progesterone, and estradiol. Prenatal/perinatal exposure to testosterone in males leads to systematic effects on the CNS and promotes the development of multiple male-typical behavioral phenotypes, including eating habits. Studies conducted on animals have shown that administering exogenous testosterone treatment during perinatal periods resulted in a significantly lower rate of propensity toward binge-eating among female rats compared to their control counterparts.^114^ In adulthood, elevated levels of testosterone may heighten the vulnerability to pathological eating behaviors in women. Patients with BN exhibited diminished symptoms of binge-purge cycles subsequent to treatment with a testosterone receptor antagonist.^115,116^ In regards to the role of ovarian hormones, early studies showed that estradiol exerted direct anorexic effects on eating behavior, whereas the stimulatory effects of progesterone were mainly indirect through its antagonism of estradiol.^117^ In addition, estradiol possesses the ability to modulate specific neural circuits associated with binge eating disorders, encompassing those implicated in the motivation for food cravings and regulation of appetite. Enhanced neuronal activity within the amygdala and periventricular nucleus of the hypothalamus has been linked to heightened levels of binge eating behavior in ovariectomized adult female rats.^118^

Of note, sex hormones and gut microbiota are believed to have a close bidirectional relationship. A study comprising 1135 participants revealed that the gut microbiota of females exhibited substantial diversity, which could be affected by the use of birth control pills or ovariectomy.^119^ Gut microbiota riched with β-glucuronidase regulates estrogen levels through facilitating its metabolism in the enterohepatic circulation.^120^ Additionally, estrogen can shapes the composition of the gut microbiota and increases intestinal permeability through its long-lasting stimulatory effects on immune cells including dendritic cells and B lymphocytes.^121^ Considering the pivotal roles played by the gut microbiota in regulating feeding behavior, interactions between sex hormones and the gut microbiota may also contribute to gender disparities observed in the prevalence of binge eating disorders.

## 7. Gut-brain axis-based therapeutic approach for the treatment of binge eating

Currently, international guidelines recommend psychological therapies such as cognitive behavioral therapy (CBT), dialectical behavioral therapy (DBT), and interpersonal psychotherapy (IPT) as the foremost therapeutic approaches for individuals with binge eating disorders, including both bulimia nervosa (BN) and binge eating disorder (BED). For the treatment of adult BN, it is recommended to utilize BN-focused self-help programs that incorporate cognitive-behavioral materials and include brief supportive sessions. When addressing child and adolescent BN, the primary treatment option lies in employing BN-focused family therapy. As for individuals with BED, both adults and children, it is recommended to follow a BED-focused guided self-help program that incorporates cognitive-behavioral materials along with brief supportive sessions.^122^ These interventions primarily focus on attempts to increase self-control and reduce binge eating episodes.^123,124^ The evidence of efficacy for CBT is the most extensive among these approaches, and it can be easily adapted into scalable formats such as self-help-only or guided interventions.^125^ The efficacy of CBT in reducing the frequency of binge eating episodes for BED treatment is particularly remarkable, despite its limited impact on weight loss.^126,127^ Pharmacological interventions for the treatment of binge eating include anticonvulsants (such as zonisamide and topiramate), anti-obesity medications (like orlistat), antidepressants (including selective serotonin reuptake inhibitors, SSRI) and CNS stimulants (such as lisdexamfetamine, LDX).^8^ The SSRI is beneficial in terms of patient acceptance as well as reducing binge symptoms.^128^ The LDX stands as the sole FDA-approved medication for the treatment of moderate to severe BED in adults within the United States, while its use in other countries remains out of indication.^129^ Meta-analyses suggest that both the SSRI and the LDX exhibit remarkable efficacy in mitigating or eradicating episodes of binge eating among patients with BED when compared to placebo,^126,130,131^ yet the impact of medication on the mood and psychopathology of binge patients has yield inconsistent results. Available clinical data on SSRIs indicate that the remission of binge symptoms is no longer substantial after 3 to 6 months.^66^ Another review, based on current evidence, posited that the amalgamation of pharmacological interventions with psychological therapies may yield superior outcomes compared to monotherapies in binge patients with comorbidities, although only 4 of 12 included trials lend support to this proposition.^132^

Improved understanding of dysfunction in the gut microbiome and the gut-brain axis has prompted the exploration of a microbiome-based intervention approach for the treatment of EDs. Terry et al. recently completed a critical analysis of the gut microbiome’s function in relation to EDs. The authors arrived at the conclusion that augmenting the population of gut microorganisms such as *Bifidobacterium* spp., *Lactobacilli* spp., and Enterococcus spp. would likely ameliorate symptoms associated with EDs.^133^ Meanwhile, the composition of the gut microbiome can be modified through procedures such as fecal microbiota transplantation, administration of antibiotics, supplementation with prebiotics and probiotics.^24^ The FMT is currently considered as a promising therapeutic option for patients with mental disorders including EDs. de Clercq et al. reported a case wherein FMT from a healthy donor to an patient with anorexia nervosa resulted in substantial weight gain by enhancing the production of SCFAs and enriching the abundance of beneficial microbiota communities.^134^ Prebiotics and probiotics supplementation has demonstrated efficacy in alleviating symptoms and improving gastrointestinal functions in patients with inflammatory and functional bowel disease.^135,136^ Bifidobacteria, lactobacilli, Enterococci are the main types of probiotics essential for the synthesis of SCFAs, production of neurotransmitters including GABA and 5-HT, stimulation of the immune system and cytokine release, as well as augmentation of the intestinal barrier function.^137^ Studies on both mice and human volunteers have revealed a notable surge in the occurrence of neuropsychiatric disorders subsequent to gut microbiota dysbiosis, and a significant decline following oral administration of probiotics.^138,139^ Up to now, clinical trials investigating the effectiveness of gut microbiota transplantation and probiotic supplementation in BED and BN patients are very lacking. In a randomized controlled trial, 101 patients were treated with either specific probiotics (i.e., Lactobacillus and Bifidobacterium) or placebo supplements for 90 days after bariatric surgery. The findings demonstrated that the utilization of probiotic treatment exhibited a significant positive impact on ameliorating binge eating scores and mitigating symptoms associated with food addiction, even after the passage of 1-year post-surgery, when compared to the control group receiving placebos.^140^

Our latest study, utilizing 16s rDNA sequencing and metabolomic analysis, has revealed a dramatic decrease in *Faecalibacterium prausnitzii* and KYNA levels within the gut of BN patients. Notably, colonization of *Faecalibacterium prausnitzii* within the intestines of binge-model mice led to significantly alleviated overeating behaviors, akin to those observed following oral supplementation of KYNA.^12^ Recent data on the psychological advantages of using probiotics to optimize the gut microbiota further suggests that microbiota-based treatments can yield benefits for individuals with eating disorders in terms of ameliorating symptoms of anxiety and depression.^141,142^ These findings, in conjunction with prior investigations, strongly suggest the auspicious potential of microbiota and gut-brain axis interventions in the management of binge eating disorders. Therefore, a crucial subsequent step entails the identification of pivotal bacterial strains or groups of metabolites that consistently contribute to the pathology of binge eating, followed by the further development and implementation of large-scale clinical trials involving prebiotic or probiotic supplementation in individuals with BED and BN. This will facilitate the establishment of evidence-based interventions targeting gut microbiota, ultimately benefiting patients afflicted with binge eating disorders.

## Data Availability

This review does not address original research data that need to be publicly deposited.
